# Nutritional Management of Patients with Fontan Circulation: A Potential for Improved Outcomes from Birth to Adulthood

**DOI:** 10.3390/nu14194055

**Published:** 2022-09-29

**Authors:** Letizia Baldini, Katia Librandi, Chiara D’Eusebio, Antonella Lezo

**Affiliations:** 1 Postgraduate School of Pediatrics, University of Turin, 10126 Turin, Italy; 2 Pediatria Specialistica, Ospedale Infantile Regina Margherita, Piazza Polonia 94, 10126 Torino, Italy; 3 Dietetic and Clinical Nutrition Unit, Pediatric Hospital Regina Margherita, University of Turin, 10126 Turin, Italy

**Keywords:** Fontan circulation, nutrition, faltering growth, congenital heart defects, univentricular heart

## Abstract

Fontan circulation (FC) is a surgically achieved palliation state offered to patients affected by a wide variety of congenital heart defects (CHDs) that are grouped under the name of univentricular heart. The procedure includes three different surgical stages. Malnutrition is a matter of concern in any phase of life for these children, often leading to longer hospital stays, higher mortality rates, and a higher risk of adverse neurodevelopmental and growth outcomes. Notwithstanding the relevance of proper nutrition for this subset of patients, specific guidelines on the matter are lacking. In this review, we aim to analyze the role of an adequate form of nutritional support in patients with FC throughout the different stages of their lives, in order to provide a practical approach to appropriate nutritional management. Firstly, the burden of faltering growth in patients with univentricular heart is analyzed, focusing on the pathogenesis of malnutrition, its detection and evaluation. Secondly, we summarize the nutritional issues of each life phase of a Fontan patient from birth to adulthood. Finally, we highlight the challenges of nutritional management in patients with failing Fontan.

## 1. Introduction

Fontan circulation (FC) is a palliation state which represents the end stage (pre-transplant) of different types of congenital heart conformations that are classified as functional univentricular hearts. Almost ten percent of congenital heart defects (CHDs) are classified as functional univentricular [1]. This wide range of cardiac malformations varies from hypoplastic left heart syndrome (HLHS) to hypoplastic right ventricle and unbalanced atrioventricular septal defect. The more known palliative-staged surgery includes a Norwood procedure (or hybrid) in the first week of life, superior cavopulmonary connection (SCPC) at 3–6 months, followed by a total cavopulmonary connection (TCPC) by Fontan procedure at 2–5 years of life [2]. Thus, we recognize a pre-stage 1 (before Norwood procedure), an interstage 2 (before SCPC), and an interstage 2 (before TCPC).

In recent years, the fetal and perinatal management of HLHS has improved, leading to a prenatal detection rate ranging from 39 to 97% [3,4,5]. Moreover, the surgical technique has evolved into the lateral tunnel and extracardiac conduit approaches, resulting in improved survival and a decreased rate of complications [6,7]. Even if approximately one-third of live-born neonates with HLHS still die before any surgical intervention [8], according to a recent systematic review [9], the 15-year survival rates reached up to 95% during the most recent surgical era, compared to 52–82% of earlier reports on patients with FC [10,11,12,13]. Nevertheless, there is increasing evidence of significant comorbidities in survivors involving multiple organ systems, which undoubtedly impact the quality of life and mortality of this population [14]. Malnutrition is a matter of concern in children with CHDs, leading to a longer hospital length of stay, a higher risk of infection, higher mortality rates, a higher risk of adverse neurodevelopmental outcomes, and ongoing family stress [15,16,17,18]. Notably, it has been shown that a lower weight-for-age Z-score (WAZ) correlates with interstage morbidity in single ventricle (SV) patients and is an independent and potentially modifiable risk factor for stage 2 complications [19,20]. In addition, feeding issues and impaired growth are significant predictors of adverse neuro-developmental outcomes in these patients [21]. Conversely, it has been demonstrated on a population of 148 infants that normal interstage growth and WAZ may be achieved through the close surveillance of nutritional status, and it is associated with excellent interstage survival [22].

## 2. Methodology

**Figure 1:**
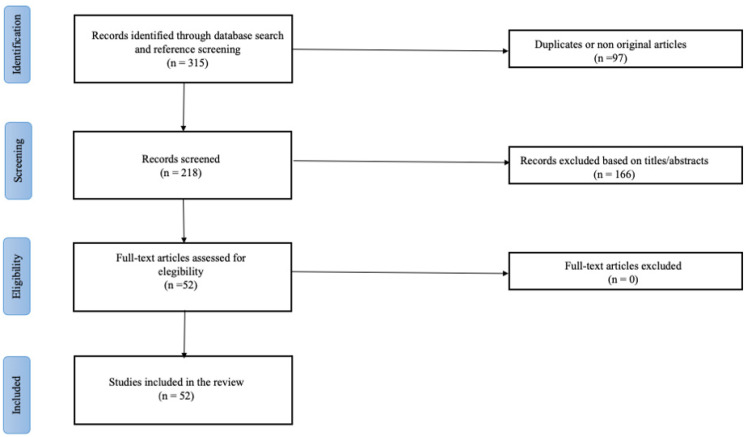
PRISMA-chart summarizing search and selection process.

This narrative review was conducted according to the Preferred Reporting Items for Systematic Reviews and Meta-Analysis (PRISMA) guidelines. We performed a comprehensive search through PubMed (National Library of Medicine); EMBASE (Excerpta Medica database); and Cochrane Library, combining the keywords “Fontan circulation” OR “single ventricle” with “nutrition therapy” OR “nutrition assessment” OR “growth”. MeSH (Medical Subject Headings) was used to enhance the search process. All the relevant articles published and written in the English language were included, and preference was given to the sources published within the past 15 years. The results of the search and selection process are summarized in Figure 1. We also referred to evidence-based guidelines regarding Parenteral Nutrition of Children and Pediatric Critical Care Nutrition, such as the European Society of Pediatric and Neonatal Intensive Care (ESPNIC) recommendations for CHD newborns, and the Guidelines for the Provision and Assessment of Nutrition Support Therapy in the Pediatric Critically Ill Patient (ASPEN) [23,24]. Every article was screened by at least two reviewers, who worked independently. The results were organized and discussed according to their subject. The suggestions and opinions expressed in this review are also based on personal experience in the management of patients with FC.

## 3. The Burden of Faltering Growth in Patients with Fontan Circulation

### 3.1. Epidemiology

FC is the common end stage of different CHDs, which implies different comorbidities, neuromotor abilities and, thus, different preoperative nutritional conditions. Due to its clinical variety, a severity stratification of malnutrition in this population is difficult. Nevertheless, all patients with univentricular heart share significant nutritional deficits, starting from the early neonatal phase and continuing with growth [25]. A retrospective study on 231 patients who had undergone Bidirectional Cavopulmonary Shunt (BCPS) and Fontan procedures highlighted a failure to tolerate or achieve sufficient caloric intake via oral feeding in 42 (33%) patients in the pre-BCPS period, 22 (18%) patients in the pre-Fontan period, and six (5%) patients in the post-Fontan period [26].

A longitudinal analysis of a cohort of 555 patients with SV showed that the greatest drop in WAZ was observed between birth and Norwood discharge, with a steady gradual decline through the interstage period to the stage 2 procedure, followed by the period of highest gain occurring between stage 2 and 14 months of age [25].

### 3.2. Pathogenesis

Major factors contributing to growth retardation in infants with SV include inadequate caloric intake, high metabolic demand [27,28], hypoxemia [29,30], altered gastrointestinal physiology, and coexistent genetic and extracardiac abnormalities.

First, unattended high metabolic demand is unavoidably related to malnutrition or faltering growth: indeed, variables associated with a more complex postoperative course and imbalance between systemic and pulmonary blood flow are associated with poorer nutritional status [19]. Thus, the preservation of heart function during the earliest phases may allow for adequate subsequent catch-up growth [31]. Admittedly, it appears that early surgical intervention leans in favor of adequate catch-up growth [32,33].

The need for pharmacological therapy, notably ACE-inhibitors, is also associated with impaired growth [26,34]. Furthermore, due to the immaturity of the gastrointestinal tract, poor digestion, and limited chewing ability, in children with FC, feeding difficulties, specific food preferences, and antifeeding behaviors are significantly more frequent [35,36]. Finally, faltering growth in patients with FC may be due to a genetic syndrome eventually associated with CHDs [37]; an analysis on 503 patients undergoing Fontan palliation identified chromosomal abnormalities in 107 neonates, which included Down, Turner and Klinefelter syndromes, heterotaxy syndrome, and others [38].

Even so, we can count on nutritional strategies adopted during early hospital stay to significantly improve growth. It has been demonstrated that infants undergoing cardiac surgery while receiving a more aggressive feeding intervention have significantly better weight gain and improved clinical outcomes [39], and have higher WAZ at discharge [19].

## 4. Assessment of the Nutritional Status

**Figure 2:**
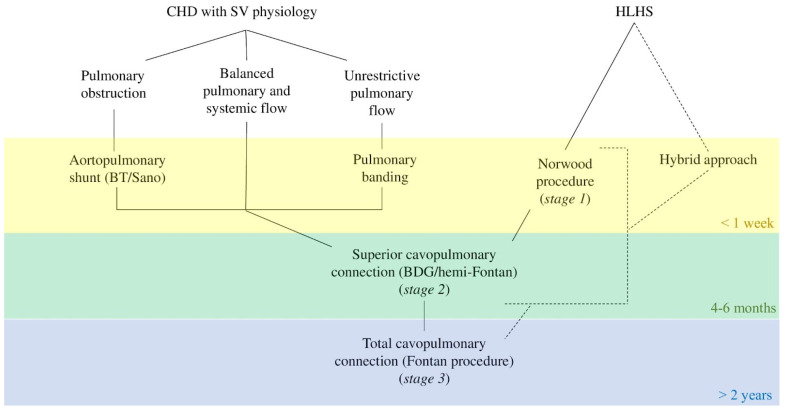
Scheme of more common surgical stages ending with Fontan procedure. Abbreviations: BDG, bidirectional Glenn; BT, Blalock–Taussig; CHD, congenital heart defects; HLHS, hypoplastic left heart syndrome; SV, single ventricle.

Regardless of the stage of the surgical journey (which is summarized in Figure 2), the assessment of the nutritional status should be based on anthropometry, as well as body composition measurement. Nutritional intervention should start from the definition of energy requirements and dietary intake. Measurements of weight, height, and head circumference in infants, which must be taken using standardized techniques and calibrated equipment, as well as Body Mass Index (BMI) calculation, are essential to properly assess growth, nutritional status, energy requirements, and for monitoring nutritional interventions [40]. To plot individual measurements, the 2006 WHO charts should be used for children up to 2 years of age, while CDC 2000 charts should be used for children and adolescents aged 2–20 years. Monitoring Z-scores is advisable in clinical practice [41]. When a genetic syndrome coexists, appropriate growth curves should be applied (i.e., curves for children with Down syndromes and CHDs; curves for girls with Turner syndrome) [42,43]. The BMI Z-score is used especially as an indicator of overweight and obesity in children, while appropriate growth and nutritional status are defined through the Z-score of weight and height for age. Indeed, acute malnutrition is defined as ponderal deficit (wasting) expressed by WAZ or BMI-for-age Z-score < −2, while >-score < −1 refers to mild malnutrition or at-risk condition [44]. Stature deficit (stunting) may be a consequence of chronic malnutrition expressed by height/length-for-age Z-score < −2. Nonetheless, weight, height, and BMI cannot return information on body composition, such as the percentage of body fat and lean mass, which have different metabolic and clinical significance. Techniques for determining body composition are skinfold thickness (subject to high rates of inter-observer and intra-observer errors); mid-upper-arm circumference (MUAC); bioelectric impedance analysis; and dual-energy X-ray absorptiometry (usually used in research settings).

Nutritional assessment should be completed by a physical examination, searching for signs of malnutrition such as depleted subcutaneous fat, muscle wasting, dry skin, and blood tests. Biochemical signs of malnutrition include low levels of total plasma proteins, serum albumin, thyroxine binding globulin, prealbumin, transferrin, ceruloplasmin, retinol-binding protein, lymphocytes, water-soluble (B2, B12, B9, C) and liposoluble (A, D, E) vitamins, and trace elements (copper, selenium, zinc, iron, calcium) [40]. Moreover, patients with FC present a significant risk of reduced levels of antioxidants such as selenium, zinc, and ascorbic acid, because of their high level of oxidative stress. Maintaining iron within normal levels is especially important for cyanotic individuals, while determinations of total cholesterol, HDL, and triglycerides are useful to assess cardiovascular risk [45]. All the aforementioned deficiencies must be promptly detected and adequately corrected by means of supplementation and/or the optimization of nutritional intakes. Shifts in blood tests and the determination of body compartments are useful tools for monitoring the nutritional intervention [44].

As far as energy requirements are concerned, the gold standard for basal metabolic rate determination is indirect calorimetry. However, since this is often impractical, basal energy requirements are more frequently estimated through calculation, i.e., by the equations endorsed in the 1985 report of the FAO/WHO/UNU expert consultation [46]. Useful tools for the determination of dietary intakes are 24-h dietary recall and quantitative food diaries (of 3–7 days) [40]. Given the specificity and complexity of these patients, referral to an experienced Pediatric Nutrition Team is advisable both during hospital stay and after discharge, as the team is responsible for the long-term monitoring of growth, body composition, and the efficacy of nutritional intervention.

## 5. Nutritional Management up to Fontan Surgery

### 5.1. From Birth to Stage 1

The most common surgical Stage 1 approach is the Norwood procedure: reconstruction of the aorta using the ascending aorta and native main pulmonary artery, in order to ensure an appropriate pulmonary blood flow through a modified Blalock–Taussig shunt (subclavian or innominate artery and pulmonary arteries) or a Sano shunt (right ventricle to pulmonary artery) and guarantee an unobstructed left atrial egress via atrial septectomy. A valid alternative to the Norwood procedure is a hybrid technique which integrates surgery and interventional catheterization. At birth, SV patients’ weight is usually adequate to gestational age, though lower than the mean of the general population, with a median Z-score ranging from −0.7 to −0.01, as suggested by some retrospective studies; several studies have demonstrated a significant drop in anthropometric measures in the early neonatal period [25,26,37], which is consistent with other reports on children with CHD [47]. In fact, meeting the nutritional requirements is often not feasible from pre-stage 1, and most newborns with a functional univentricular heart do not receive enteral nutrition. In addition, the numerous surgical interventions and hospitalizations cause abrupt interruptions of a delicate phase of growth and neuromotor development, including the oral-motor learning and training parts [48]. Early nutritional management is controversial, primarily because of the association between early enteral feeding and necrotizing enterocolitis (NEC) in newborns with CHDs [49], notably those with SV physiology and/or shunt-dependent pulmonary blood flow [50,51,52]. The risk of NEC has often led to a prohibitive approach to enteral nutrition (EN) (mean time to the initiation of EN is 6 days, and 84% of infants not receiving any EN preoperatively) [37], significant differences in feeding practices, and the emergent necessity of a standardization of the latter. A feeding algorithm for patients destined to Fontan palliation has been elaborated, showing a significantly less negative WAZ change from birth to hospital discharge after implementation of the postoperative feeding algorithm [52]. However, the most challenging phase is the critical post-stage 1 surgery, where malnutrition is often associated with postoperative complications: delayed sternal closures, vocal cord palsy, and chylothorax might hamper the provision of adequate nutrition in this phase [23,53,54,55]. Aware of the adverse impact of feeding difficulties on these patients [56,57,58,59], the main factor determining a reduction in delivered EN remains fluid restriction [60]. Low cardiac output, a transient state of renal impairment or acute kidney injury after cardiopulmonary bypass [61], as well as the use of numerous postoperative infusions, adversely affect the volume destined to TPN or EN. Furthermore, these patients are not always liberalized to full maintenance fluid therapy immediately after surgery, and the goal of postoperative PN in full term newborns is rarely achieved in these patients [62]. Aside from the severity of postoperative course, the use of PN and high calorie enteral feeds is associated with improved nutritional status, and it seems that aggressive parenteral and EN therapy after stage 1 surgery might help reduce the prevalence of growth faltering in infants with HLHS [19]. At the time of initial discharge home from stage 1 surgery, between 25% and 75% of patients with SV require home EN [16,19,57].

### 5.2. From Interstage 1 to Stage 2

After the Norwood procedure, i.e., in a context of univentricular circulation, the SV pumps to great vessels placed in parallel (the aorta and the pulmonary arteries). As a result, pulmonary blood flow is excessive, and the right ventricle works at systemic pressures and high-volume load, from both the pulmonary and systemic circulations. The load of pressure and volume of the RV leads to its progressive dilation, which requires a load reduction through the second stage. Thus, the palliation continues with the SCPC, including the bidirectional Glenn (end-to-side anastomosis of the superior vena cava to the right pulmonary artery) and the hemi Fontan procedure, which is an anastomosis of the confluence of the superior vena cava and the right atrium to the branch pulmonary artery using a homograft dam to redirect the flow of the superior vena cava to the pulmonary arteries. The bidirectional Glenn, as well as the hemi-Fontan, unloads the SV by the removal of the venous return (preload) from the upper body. The result is a reduction in RV volume, its wall stress, and afterload. Stage 2 prevents the further deterioration of RV ejection fraction.

Currently available data regarding growth during interstage 1 are controversial, with some reporting a continued loss of WAZ [25] and others showing a trend toward improved WAZ between post-stage 1 discharge and stage 2 surgery [37]. Nonetheless, this period is particularly risky, with reported mortality rates as high as 22% [22,63,64]. In a retrospective study on 124 patients with FC, factors impairing growth in the period pre-BCPS included the number of surgical procedures and higher right atrial pressures at catheterization. Moreover, the presence of two or more active venous collaterals was associated with impaired growth independently from arterial oxygen saturation and pulmonary artery pressure [26]. Growth is known to be an important sign of good health, so ensuring an adequate growth rate during this critical period is crucial, as it is associated with improved operative and long-term outcomes [22].

### 5.3. From Interstage 2 to Stage 3

Stage 3 is represented by the TCPC. The Fontan operation completes the separation of pulmonary circulation from systemic. The procedure can be performed in two ways: one is the lateral tunnel Fontan, incorporating an intra-atrial conduit from the inferior vena cava to the pulmonary artery; the second is the extracardiac Fontan, with a conduit that connects the inferior vena cava to the pulmonary arteries, avoiding atrial distension and arrhythmias. Following stage 2 surgery, up to 22% of patients fail to achieve adequate caloric intake, requiring feeding tube supplementation [26,35]. However, as seen in other CHDs requiring surgical correction [47], weight usually improves by the time of completion of the FC, and for the following first two years. Nonetheless, some authors reported a failure to achieve adequate caloric intake requiring feeding tube supplementation in 5% of patients following Fontan completion, and in long term follow-up, both mean weight and length for age Z-scores remain significantly lower than the mean of the normative population, as well as healthy parents and siblings [25,26,65].

### 5.4. General Approach up to Fontan Surgery

Despite the peculiarities of patients with HLHS or SV, in consideration of their vulnerability and in the lack of specific guidelines, it seems reasonable to follow the European Society of Pediatric and Neonatal Intensive Care (ESPNIC) recommendations for CHD newborns, and the Guidelines for the Provision and Assessment of Nutrition Support Therapy in the Pediatric Critically Ill Patient (ASPEN) beyond the neonatal period [23,24].

It Is recommended to perform a detailed nutritional assessment on admission and at least weekly during hospital stay and after, and to express these measurements in Z-scores, including weight, height/length MUAC, and head circumference in young children, considering that weight could be affected by fluid imbalance. In the acute phase, energy intake provided to critically ill children should not exceed resting energy expenditure, while after the acute phase, energy intake provided to critically ill children should account for energy debt, physical activity, rehabilitation, and growth [23,24]. Daily measurements of resting energy expenditure using indirect calorimetry and nitrogen balance is the best way to optimize energy and protein intakes, avoiding under or overfeeding [66]. EN is also recommended in stable patients receiving hemodynamic pharmacological support or extracorporeal life support. However, it is suggested to progressively achieve at least two-thirds of the prescribed daily energy requirement by the end of the first week in the PICU, with adequate amounts of protein, in order to reach better clinical and nutritional outcomes [24]. Slicker et al. proposed a nutritional algorithm for infants with HLHS from birth through the first interstage period [67]. The authors suggest that enteral feeding is safe in hemodynamically stable patients under an appropriate level of monitoring. Moreover, PN should be initiated if the EN goal is not achieved and advanced to full calorie and protein goals. Nevertheless, the initiation of PN within 24 h of PICU admission is not recommended and it may be postponed up to one week, while providing trace elements and vitamins, if EN is feasible [23,24]. Furlong-Dillard et al. suggested that a standardized nutritional approach in this phase can increase the percentage of patients enterally fed before surgery and reduce the postoperative use of TPN, without an increase in complications [68]. At any rate, the use of a stepwise algorithmic approach is recommended to advance EN in children admitted to the PICU, including bedside support to guide the detection and management of EN intolerance and the optimal rate of increase in EN delivery [24].

Oral feeding must also be promoted, as the literature highlighted that patients who are orally fed during the preoperative period were more likely to be fed orally at hospital discharge [52], and preoperative trophic/minimal enteral feeding may prime the intestine to tolerate enteral feeding after surgery [37]. In hemodynamically stable term neonates with or without pharmacological cardiovascular support, EN should be started within 24 h from admission [23]. EN is also the preferred mode of nutrient delivery to the critically ill child [24]. When oral feeding is possible, breast milk should be preferred in newborns and infants because of its relevant role in reducing the risk of NEC [53].

There is evidence that the choice of feeding method at admission for stage 2 palliation has a significant long-term impact, so particular attention should be paid to this matter. Although tube feeding may help achieve caloric needs, oral feeding should be encouraged, as feeding methods are not likely to change by discharge, and also impact on length of stay [69].

## 6. Nutritional Management after Fontan Surgery

### 6.1. Energy Requirements

Patients with FC show a wide range of energy requirements, depending on several factors such as: the type and severity of CHD, age at the time of intervention, and time since FC. Following the Fontan operation, the SV pumps to the systemic and pulmonary circulations are placed back in series, without a sub pulmonary pump. This implies a striking increase in the central venous pressure. However, the FC is an effective palliation for patients with an SV. Indeed, patients who underwent the Fontan procedure showed a stabilization of WAZ [26]. However, they are often wasted [70] and stunted [37] and an extra supply of energy is required for catch-up growth. Notably, children affected by acyanotic lesions tend to be wasted because of the fluid buildup in the lung airspaces that increase respiratory effort, while children with cyanotic lesions tend to be stunted because of the low level of oxygen in the blood [71,72,73,74]. Obviously, children with pulmonary hypertension and cyanosis show the greatest nutritional risk [71].

Energy requirements also depend on disease course after intervention. Following the eventual catch-up-growth [26], energy expenditure seems to equalize peers’ requirements. Moreover, factors such as relief of food-related symptoms, low levels of physical activity due to impaired aerobic capacity [75,76] and, eventually, the presence of stunting [77], may provoke excess in weight gain. Indeed, overweight and obesity involve 15% to 35% of patients with FC [78].

For all these reasons and based on the age at intervention (ranging from 2 to 10 years), energy requirements vary widely: it is recommended to refer to the caloric needs of healthy peers and then adjust based on the trend of growth and on BMI Z-score. Thus, regular nutritional assessment is strongly encouraged in these patients. Due to the extreme variability, whenever possible, energy requirements should be defined and monitored through indirect calorimetry. Nutritional intervention should, therefore, be driven by the assessed gap that exists between the energy requirements and the food nutritional intake [40].

In addition, we suggest following up stable patients on an annual basis and monitoring weight, height, and BMI Z-score, as well as serum albumin, total protein, calcium, iron, selenium, vitamin A, 25-OH Vitamin D, and vitamin E; energy requirements and dietary intake should be periodically re-evaluated and tailored to the patient’s needs.

### 6.2. Somatic Growth and Bone Mineralization

Both mean weight and length for the age Z-scores of patients with FC remain significantly lower than the mean of the normative population and their parents and siblings, despite the fact that many patients are within normal limits for height and weight [25,79]. The specific pathophysiology of how FC affects somatic growth is still unclear, although the deficit in linear height has been associated with diminished functional health status [79]. Although the precise mechanism by which low cardiac output and central venous hypertension affect the bone structure is still unclear, an association between the cardiocirculatory milieu of FC and bone abnormalities has been demonstrated; particularly, lower cortical area (−0.59 ± 0.84 vs. 0.00 ± 0.88, *p* < 0.001) and periosteal circumference (−0.50 ± 0.82 vs. 0.00 ± 0.84, *p* < 0.001) Z-scores were highlighted in a cohort of 43 patients by Avitabile et al. [80]. Furthermore, secondary hyperparathyroidism is common in FC, due to the alterations in calcium metabolism resulting from changes in renal perfusion and poor intestinal absorption. The parathyroid hormone may be a valuable biomarker of heart failure and may be helpful in predicting outcomes in patients with FC [81]. Finally, there is a relationship between the mediators of growth hormone and circulating levels of serum brain natriuretic peptide (BNP) [80], suggesting that an adequate treatment of heart failure could reverse the decline of the growth factor, improve bone health, and promote longitudinal growth.

Many studies have highlighted a high percentage of patients with FC with decreased bone mineral density (BMD), as shown by dual X-ray absorptiometry or significant bone deficits revealed by peripheral quantitative CT [80,82,83,84,85,86,87]. Indeed, hypoxia is a promoter of osteoclast differentiation and bone reabsorption [86]. Moreover, the multifactorial etiology and high incidence of hypovitaminosis D, which in some cohorts of FC patients reaches 70% [88], further impairs BMD. Children with PLE are at particular risk for reduced BMD because of chronic hypocalcemia that is secondary to low levels of albumin [83]. In a recent study, 42% of the subjects showed BMD below −2 SDs, and the mean serum calcium level of the entire cohort appeared to be below normal range. Interestingly, patients with both a height and weight Z-score below average showed lower DXA Z-scores, highlighting the direct correlation of nutritional status and bone health [89]. More severe bone disease is reported in patients with FC and PLE, due to chronic hypocalcemia linked to chronic hypoalbuminemia and enteral losses.

### 6.3. Altered Body Composition

The body composition of patients with FC is characterized by a deficiency of skeletal muscle mass [85,90,91] to such an extent that the term Fontan-associated myopenia has been suggested to describe significant muscle deficits (lean body mass Z score <−2). In a study conducted on a cohort of adolescents with FC [92], the percentage of skeletal muscle mass appeared to be associated both to having suffered from late complications of FC intervention and to lower peak oxygen consumption. Moreover, low skeletal muscle mass has been linked to reduced exercise capacity, oxygen pulse, lower ventricular systolic function, and compensatory erythrocytosis [85]. While some factors leading to reduced muscle mass are not modifiable, others, such as adequate protein intake and physical activity, may be achieved with appropriate management [93].

Conversely, thanks to the advance in care and the subsequent increased life expectancy of children who undergo FC, new issues are rising regarding their nutritional management. Low energy expenditure due to low skeletal mass, exercise intolerance, and frequent hospitalizations may lead to an increased risk of being overweight or obese if nutritional follow-up is not performed and/or in the case of inappropriate dietary intake. Moreover, in the presence of low skeletal muscle mass, the sole BMI may be misleading and underestimate fat mass. The US “Fontan rehabilitation, wellness and resilience development”, or FORWARD program (path forward), claims that a specific “Fontan diet” does not exist. Instead, for most patients without growth deficit, the recommendation is for a diet high in fruits, vegetables, whole grains, fiber, unsaturated fat, and omega 3 fatty acids, and low in fat dairy, added sugars, sodium, and saturated/trans fats. Indeed, this type of diet is proven to optimize cardiovascular health [94]. It is of detrimental importance to prevent obesity in people with FC, as a higher BMI has been associated with symptomatic heart failure and higher mortality in a cohort of adult patients [95]; in fact, obesity may worsen chronic myocardial dysfunction and/or residual anatomic lesions and increase the risk of Fontan failure [96]. For these reasons, we suggest regularly monitoring MUAC and performing a bioimpedance analysis during follow-up.

### 6.4. Protein-Losing Enteropathy

PLE is defined as the presence of hypoalbuminemia (<30 g/L) with no other identifiable cause of protein loss other than the gastrointestinal tract [97], occurring after Fontan palliation in 5–12% of patients [23,53,97,98]. The morbidity and mortality of this condition are still significant [99,100,101]. Signs of PLE appear after a median interval of 3.7 years (range 1.2–9.7) from Fontan intervention [102].

Even if its cause is not fully understood, recent literature reported an increase in abnormal lymphatic vessels throughout the intestinal tract [103], as well as a lymphatic congestion typically causing leakage and protein loss into the intestinal tract [100,103,104]. Cardiac dysfunction is a worsening factor, as low cardiac output causes blood flow redistribution, with a selective increase in mesenteric vascular resistance [105], as well as a proinflammatory state causing the alteration of enterocyte membrane permeability [106,107]. Some patients with PLE present with reduced heparan-sulfate proteoglycans, enterocyte membrane proteins involved in protein trafficking whose absence can cause protein leakage [106]. Essentially, the decrease in serum oncotic pressure causes systemic oedema, and the intestinal wall oedema worsen malabsorption, thus, perpetuating the vicious cycle of protein loss in episodes called PLE flares.

The gold standard for the diagnosis of PLE Is the elevation of fecal α-1- antitrypsin, but false negatives (in the case of loss from the stomach) and false positives (in the case of diarrhea) have also been observed [97]. In clinical practice it is important to monitor early signs of PLE, such as low serum albumin and total proteins that drive the diagnosis of PLE, although studies showed that enteric protein loss begins before the appearance of hypoproteinemia [107,108].

Other blood anomalies often encountered in patients with PLE are low plasma levels of fat-soluble vitamins (A, D, E); calcium (linked to chronic shortage of serum albumin and intestinal losses); and immunoglobulins. The deficiency of Vitamin D and calcium place children at a higher risk of low BMD [83], whilst poor antioxidant status due to low vitamin A and E impair the red-ox balance and predispose to tissue damage by free oxygen radicals. Thus, monitoring vitamin status is crucial, and in the case of vitamin insufficiency, supplementation is required. Blood determinations of liver function and urinalyses can help rule out impaired protein synthesis and renal protein losses, as in the case of nephrotic syndrome. Other causes of gastrointestinal albumin losses should also be excluded [97]. Given the possible malnutrition origin of low albumin and total protein level, accurate nutritional evaluation is recommended to establish the nature of these alterations. Clinical manifestations of PLE include chronic diarrhea, abdominal pain, ascites, peripheral oedema, and pleural effusions, but also growth failure [97]. Non-specific symptoms such as dyspnea or fatigue are also common [109].

PLE dietetic treatment is quite different from that of chronic malnutrition, being based on a multidisciplinary approach (diet, drugs, and surgery). Well-conducted trials on the impact of nutritional management on PLE are lacking and strong pieces of evidence are not available. However, due to the low cost and safety, nutritional interventions should always be part of PLE management: a high protein (>2 g/kg/day), low-fat (<25% energy intake) and normo-hypercaloric diet is recommended. Supplementation with medium-chain triglycerides (MCTs) is advisable to increase the overall energy intake. A low amount of long-chain triglyceride fats (LCTs) allows the reduction of intestinal lymphatic production and losses; instead, MCTs, due to their simple chemical structure, can be absorbed directly into the bloodstream, bypassing the lymphatic system. A low-fat diet must be managed by a specialized dietitian, avoiding malnutrition risk, and promoting compliance. If dietetic modification is unsuccessful, supportive PN may also be needed in a small percentage of patients [40,45].

## 7. Fontan Failure and Heart Transplant

Even if FC supports these patients’ lives for several years, all of them are affected by chronic heart failure, as the palliative circulation cannot ensure the metabolic requirements for blood and oxygen—at rest and during exercise—at low filling pressures [110,111]. Fontan circulatory failure is the expected result of chronic systemic venous hypertension and low cardiac output [112], which entails an exacerbation of signs and symptoms of venous congestion, such as ascites, PLE, and liver cirrhosis. Consequently, heart transplant (HT) (stage 4) is the only therapy offering long-term survival. A recent meta-analysis found survival rates to be 88% immediately after HT, 78% at 5 years, 69% at 10 years, and 61% at more than 10 years of HT [113]. On the one hand, a poor nutritional status and the presence of comorbidities may preclude the HT option; on the other hand, patients who undergo HT usually show poor nutritional status due to increased energy and protein requirements, coexisting liver disease, nutrient deficiencies, and the need for hospitalization [114], which can also affect the outcome after surgery. Thus, even if a consensus on the nutritional management of these patients is still lacking, an adequate nutritional condition is crucial for their outcomes and survival.

During the pre-transplant period, the schedule of nutritional follow-up should be more frequent than usual. Indeed, a low WAZ has been associated with an increased risk of postoperative complications and 6-months mortality following CHD surgery [115].

Nutritional status and body composition should be monitored through both anthropometric measures and blood nutritional tests, and bioimpedance analysis, as previously explained. Blood tests should include short-term indices of malnutrition, such as prealbumin and cholinesterase, and long-term indices, such as serum albumin.

After the transplant, EN is recommended because it has been linked to benefits such as the maintenance of the gut mucosal barrier and the improvement of splanchnic blood flow [116,117]. However, in the case of hemodynamic instability, PN is required. PN should be prescribed by an expert nutritionist to avoid metabolic complications such as fluid overload, overfeeding, hyperglycemia, and hypertriglyceridemia [118]. The delivery of medical nutritional therapy according to standardized protocols, preventing caloric and protein deficit, has been linked to better outcomes such as the reduced prevalence of nonocclusive bowel ischemia and reduced ICU length of stay [118].

According to the aforementioned meta-analysis [113], HT patients may present impaired renal function due to both diuretic chronic therapy [119,120] and insufficient renal perfusion, while renal failure has been associated with a higher risk of death after HT (RR = 5.8). As ICU mortality has been linked to both negative and positive fluid balance [121], post-transplant management should ensure the right fluid balance. Fluid requirements vary widely based on individual situations, such as the degree of heart failure and kidney function, but amounts of 80–100 mL/kg are usually required; diuretic therapy should be evaluated on an individual basis.

In case of chyle leaks, which can occur after chest surgery [122,123,124], medical nutritional therapy should be implemented with the aim of replacing losses of calories, protein, fat-soluble vitamins, lymphocytes, immunoglobulins, and electrolytes, which are usually present in chyle and whose deficiency may lead to electrolytes imbalance, impaired immune function, and impaired wound healing [123]. Nonetheless, protein-loss compensation with a high-protein diet may lead to hyper azotemia; therefore, the optimization of nitrogen load should be considered (maximum dose of 3 g/kg of lean body mass) [114]. Medical nutritional therapy should be implemented alongside medical and surgical therapy. The first option is a low-LCT or fat-free and high-protein diet that should reduce the lymphatic flow; however, fasting and total PN may be necessary in some cases [114]. A review found that the most common interventions were total PN and MCTs administration and that there were no significant differences in the efficacy between the different nutritional approaches in resolving chyle leakage [125]. We recommend personalizing nutritional intervention on an individual basis, as the same goals may be reached through a variety of approaches.

## 8. Clinical Implications and Future Directions

Despite advances in the management of univentricular CHDs, patients with FC have a high risk of malnutrition and growth deficit, affecting development and outcomes. Although malnutrition is known to be a modifiable risk factor, specific guidelines addressing nutritional therapy and the management of patients with FC are still lacking. In this narrative review, we aim to offer a practical guide to the comprehension and management of nutritional issues in patients undergoing Fontan surgery from birth to adulthood, according to current evidence-based literature. Ideally, we aim to help physicians carry out an appropriate nutrition assessment, detect faltering growth early, and properly set up a nutritional intervention. This may lead to a more systematic and standardized approach, and eventually to improved outcomes. The key points of the suggested nutritional interventions are summarized in Table 1.

## 9. Conclusions

Malnutrition is a matter of concern at any stage of life in children with SV, leading to a longer hospital length of stay, higher mortality rates, and a higher risk of adverse neurodevelopment and growth outcomes. Thus, it represents an opportunity to improve outcomes and survival rate. Several studies have demonstrated a significant drop in anthropometric measures in the early neonatal period and during the different surgical stages. Consequently, optimal nutrition should be pursued right from birth until Fontan intervention, according to surgery complications and the patient’s hemodynamic state; EN should be started whenever possible under appropriate monitoring. After surgery completion, the role of the Pediatric Nutrition Team remains crucial: on the one hand, growth should be strictly monitored until final height is reached; on the other hand, nutritional deficits and FC complications should be detected and compensated by nutrition optimization. When HT is indicated, the Pediatric Nutrition Team should be involved in both preparing the patient for surgery and after the procedure, especially when PN is needed. Body composition may help to understand nutritional status and define the best nutritional strategy. Further cohort studies are needed to establish the best nutritional approach, and to define appropriate and standardized feeding algorithms for each stage of these patients’ lives.

## Figures and Tables

**Table 1:** Key points of nutritional interventions in patients with Fontan circulation. REE (resting energy expenditure.)

Periodic nutritional assessment
Physical examination, measurement of anthropometric parameters (height/length, weight, head circumference) and calculation of BMI/weight for length, which should be plotted on appropriate charts and expressed as Z-score. Body composition analysis by MUAC and/or bioimpedance. Evaluation of energy requirements by indirect calorimetry or Schofield equation. Evaluation of dietary intake by food diaries and/or 24-hours dietary recall. Periodic blood tests: total plasma proteins, serum albumin, thyroxine binding globulin, prealbumin, transferrin, ceruloplasmin, retinol-binding protein, lymphocytes, water-soluble and liposoluble vitamins, and trace elements (copper, selenium, zinc, iron, calcium).
Surgical phase
Detailed nutrition assessment on admission and at least weekly during hospital stay. In the acute phase, energy intake should not exceed REE; after the acute phase, energy intake should account for energy debt, physical activity, rehabilitation, and growth. Oral feeding by breast milk should be preferred whenever possible. A stepwise algorithmic approach is recommended to advance EN during PICU stay.
Chronic phase
Stable patients may be followed-up on an annual basis. Refer to caloric needs of healthy peers and adjust based on trend of growth and on BMI Z-score. Detection and supplementation of vitamins or trace elements deficits. Periodic monitoring of albumin, immunoglobulins, and fecal fats. A diet high in fruits, vegetables, whole grains, fiber, unsaturated fats, and omega 3 fatty acids, and low in fat dairy, added sugars, sodium, and saturated/trans fats is recommended. When PLE is diagnosed, a high protein (>2 g/kg/day), low-fat (<25% energy intake), and normo-hypercaloric diet is recommended.
